# The Factors Influencing Chronic Kidney Disease Incidence: Database from the Korean National Health Insurance Sharing Service (NHISS)

**DOI:** 10.3390/jcm13082164

**Published:** 2024-04-09

**Authors:** Ho-Joon Ko, Soon-Ki Ahn, Suyeon Han, Moo-Jun Kim, Ki Ryang Na, Hyerim Park, Dae Eun Choi

**Affiliations:** 1 Department of Nephrology, Chungnam National University Hospital, Daejeon 35015, Republic of Korea; goljun@cnuh.co.kr (H.-J.K.); garlic1208@cnuh.co.kr (S.H.); 2 Department of Preventive Medicine, Chungnam National University Hospital, Daejeon 35015, Republic of Korea; withspirit09@gmail.com; 3 Department of Nephrology, Chungnam National University Sejong Hospital, Sejong 30099, Republic of Korea; kimmoojun@cnuh.co.kr; 4 Department of Medical Science, Medical School, Chungnam National University, Daejeon 35015, Republic of Korea; hye05240@gmail.com

**Keywords:** chronic kidney disease incidence, estimated glomerular filtration rate, diabetes, Korea

## Abstract

**Background:** The global prevalence of chronic kidney disease (CKD) is increasing, with diabetes accounting for the highest proportion. We analyzed the influence of clinical factors on the incidence of CKD according to the renal function, primary focusing on patients with diabetes. **Methods:** We used the Sample Cohorts Database provided by the National Health Insurance Sharing Service (NHISS) in Korea. Participants aged ≥ 40 years who underwent a health checkup in 2009 were categorized into six groups based on their eGFR values (<60 mL/min, 60–89 mL/min, ≥90 mL/min) and the presence of diabetes. And all patients with CKD at 2009 screening were excluded. The participants were tracked from 2010 to 31 December 2019. The CKD incidence rate according to the eGFR values and the effect of the accompanying factors on CKD incidence were confirmed. **Results:** 148,089 people without CKD were analyzed. The CKD incidence rate was highest in those with eGFR < 60 mL/min with diabetes and lowest in those with eGFR ≥ 90 mL/min without diabetes. The CKD incidence rates were similar between the eGFR < 60 mL/min group without diabetes and the eGFR 60–89 mL/min group with diabetes. Compared to under 44 years of age, the hazard ratio of CKD incidence was 8 times higher in over 75 years of age. Men had a 1.7-fold higher risk of developing CKD than women. Current smoker, hypertension, dyslipidemia, myocardial infarction history, and atrial fibrillation and flutter increased the risk of CKD incidence. Age, diabetes, and baseline eGFR are important factors in the occurrence of CKD. As age increases, the risk of developing CKD in men increases compared to women. **Conclusions:** These results will be helpful in predicting risk groups for CKD and establishing strategies to lowering CKD incidence.

## 1. Introduction

According to data on global chronic kidney disease (CKD) epidemiology published by The Lancet in 2020, there were 697.5 million recorded cases of all stages CKD in 2017, representing a global prevalence rate of 9.1%; This reflects a significant increase of 29.3% in the global prevalence of CKD compared to 1990. In 2007, 35.8 million individuals with CKD were undergoing dialysis. Diabetes is the leading cause of CKD necessitating dialysis, accounting for 30.7% of all dialysis patients with CKD [1].

In the Korean National Health and Nutritional Examination Survey from 2011 to 2013, the prevalence of CKD in Koreans aged ≥20 years was 8.2%, and the prevalence increased annually [2]. DM was the most common contributing factor to CKD. In another study, the prevalence of CKD in Koreans aged ≥35 years was 13.7% [3], similarly with diabetes as the highest contributing factor. Based on the statistics of the Korea Health Insurance Review and Assessment service, the number of patients with CKD in Korea increased from 203,978 in 2017 to 277,252 in 2021, with an annual average of 7.97%. The annual decrease rate of eGFR value in the eGFR group measured once was previously studied, but the actual CKD incidence rate has rarely been studied, and the study was conducted only for the elderly aged ≥66 years [4].

We analyzed the impact of various clinical factors, including estimated glomerular filtration rate (eGFR) levels and the presence of diabetes, during eGFR level measurement, on futuristic incidence of CKD. We specifically focused on the CKD incidence rate of those patients with eGFR <60 at a single time point. Furthermore, we analyzed other factors that influence the incidence of CKD based on sex.

## 2. Methods

### 2.1. Data

The National Health Insurance Service (NHIS) is the governance body of the Korean health care system, a nonprofit institution that provides health insurance to Korean citizens wherein 97% of Koreans are registered. Data from the Sample Cohorts Database provided by the National Health Insurance Sharing Service (NHISS), established to provide national health information data under NHIS, was used.

The Sample Cohorts Database sampled 2% of the national population, comprising and 1 million individuals. This database contains data on social and economic qualification variables (including death and disability), status of medical resource utilization (consultations and medical checkups), and status of the clinic. NHIS conducts health checkups for policyholders either every year or every 2 years, depending on their occupation. A surrogate variable was used to anonymize patients in the NHISS Sampling Database. In our study, the eGFR values were obtained from the values presented during health checkups. Korean health checkups regulations stipulate that the eGFR should be calculated using the MDRD formula. Consent for participants was waived because this study was a retrospective cohort study. The NHISS provided the approval for the use of the database.

### 2.2. Study Population

We included those patients who were aged 40 years or older and underwent a health checkup in 2009, had an eGFR value along with linked eligibility and death data. We excluded who had been previously diagnosed CKD from this study. Also, we excluded patients with an eGFR value of 160 or higher due to the high possibility of laboratory error. We excluded young people under 40 years of age from this study because the prevalence of CKD is significantly low [2,3].

Patients newly diagnosed with CKD with diagnosis code are updated annually with Data from the Sample Cohorts Database provided by NHISS based on National Health Insurance Claim Data.

Diagnosis codes for new CKD patients are as follows:

1. Patients with insurance claim with diagnosis code N18.x (International Classification of Diseases, 10th Revision [ICD-10]; N181, N182, N183, N184, N185, and N189)
2. Patients prescribed treatment codes (O7071, O7072, O7073, O7074, O7076, and O7077) for peritoneal dialysis
3. Patients who had undergone kidney transplantation (patients with an insurance claim with diagnosis code Z940 (kidney transplantation status) and those prescribed with surgery code R3280 (renal transplantation)
4. Calculation exception codes: Patients with insurance claims because of hemodialysis (V001; hemodialysis maintained for >90 d), peritoneal dialysis (V003), and kidney transplantation (V005).

The abovementioned conditions for the identification of patients with CKD enables selection of such patients based on the clinician’s diagnosis.

The abovementioned participants were stratified into three groups based on the eGFR measured during medical checkups in 2009 (Supplementary Table S1), as follows:

Group 1: eGFR ≥ 90 mL/min/1.73 m^2^,

Group 2: eGFR 60–89 mL/min/1.73 m^2^, and

Group 3: eGFR < 60 mL/min/1.73 m^2^

**Figure 1:**
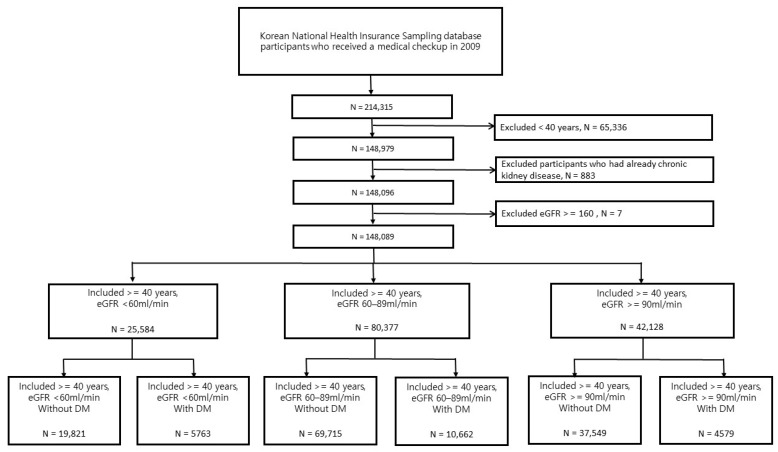
Flowchart of participant enrolment.

We further stratified the abovementioned three groups into six groups based on the presence or absence of diabetes. The flow chart shows the layering process of each group (Figure 1). After a medical checkup in 2009, the incidence rate of CKD was identified from those diagnosed with new-onset CKD from 1 January 2010 to 31 December 2019. Generally, the diagnosis of CKD is made by nephrologist, analyzing eGFR, proteinuria, and renal ultrasound in Korea. 

The participants’ comorbidities were identified as follows:

1. Hypertension: Patients with insurance claims with diagnostic codes (I10, I11, I12, I13, and I15);
2. Diabetes: Patients with insurance claims with diagnostic codes (E10, E11, E12, E13, and E14);
3. Dyslipidemia: Patients with insurance claims with diagnostic codes (E780, E781, E782, E783, E784, and E785);
4. Stroke: Patients with insurance claim with diagnostic codes (G45, G46, I63, and I64);
5. Heart failure: Patients with insurance claim with diagnostic codes (I50);
6. Atrial flutter and fibrillation: Patients with an insurance claim with diagnostic code (I48); and
7. Myocardial infarction: Patients with insurance claims with diagnostic codes (I21, I22, and I252).

Information on smoking was obtained through the medical checkup questionnaire. Income information was based on the income level provided by the NHISS Sampling Database. The 10th quintile of insurance charges was stratified into three quintiles: low (1st–3rd quintile), middle (4th–7th quintile), and high (8th–10th quintile). Medical aid beneficiaries were included in the low scale. The eGFR value was calculated using the MDRD formula (eGFR=186 × Pcr^−1.154^ × age^−0.203^ × 1.212 [if black] × 0.742 [if female]). 

### 2.3. Statistical Analysis

Continuous and categorical variables were presented as means ± standard deviation and *n* (%), respectively. The incidence of CKD grouped by eGFR and diabetes was calculated using the Kaplan–Meier curve. Multivariate Cox proportional hazards regression analysis was used to estimate hazard ratios (HRs) and 95% confidence intervals of Figures 5 and 6 shows the information on each variable adjusted for multivariate Cox proportional hazards regression analysis. All data analyses were conducted using R software (version 4.3.0; R Foundation for Statistical Computing, Vienna, Austria), and *p* < 0.05 was considered statistically significant.

### 2.4. Ethics Statement

The study was conducted according to the guidelines of the Declaration of Helsinki, and approved by the Institutional Review Board of Chungnam National University Hospital (protocol code CNUH2022-06-119 and date of approval: 4 July 2022).

## 3. Results

### 3.1. Baseline Characteristics

A total of 214,315 people who received health checkups in 2009 were analyzed. Among them, 148,089 individuals were analyzed, after excluding those under 40 years of age, patients previously diagnosed with CKD, and individuals with eGFR measured above 160. 42,128 (28%) had eGFR ≥90 mL/min, 80,377 (54%) had eGFR 60–89 mL/min, and 25,584 (17%) had eGFR < 60 mL/min. In the three groups divided based on their eGFR value, 17.3% were not diagnosed with CKD; however, their eGFR was <60 mL/min. In the group with an eGFR value <60 mL/min, there were more women than men, Cr was 1.2 ± 1.3 mg/dL, and there were significantly higher number of deaths. It was observed that the lower the eGFR, the higher was the proportion of old age and rate of having all comorbidities, including diabetes and hypertension (Supplementary Table S1).

### 3.2. CKD Incidence According to Diabetes and eGFR Levels

To determine the effect of diabetes on the incidence of CKD in the eGFR group, we had further stratified the group based on the presence or absence of diabetes. When stratified with or without diabetes, the diabetic group had a high rate of having all comorbidities, including hypertension, and the proportion of old age was high (Table 1).

**Figure 2:**
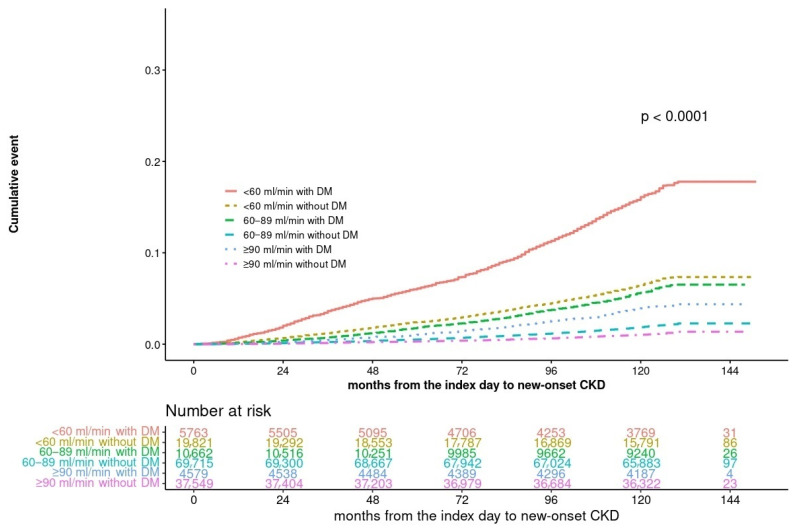
Kaplan–Meier curves for incidence of CKD by eGFR and Diabetes.

**Figure 3:**
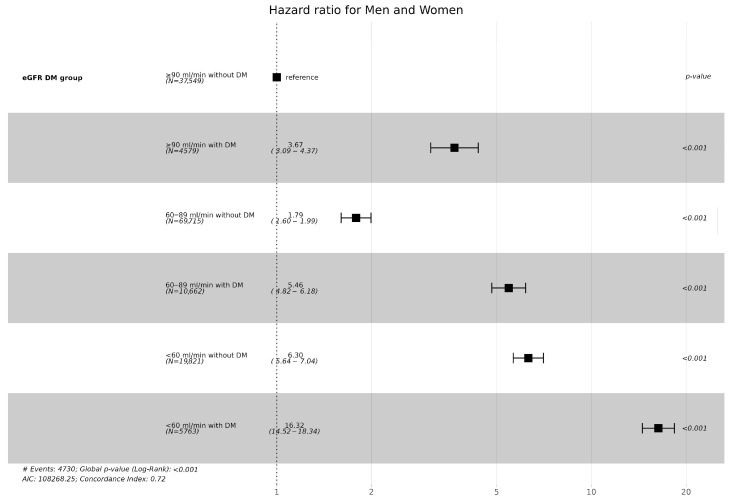
Hazard ratios (95% confidential interval) of incidence of CKD according to eGFR and Diabetes.

The incidence of CKD was the highest in the group with diabetes and the eGFR <60 mL/min. In the same eGFR group, the incidence of CKD was higher in the group with diabetes than that in the group without diabetes. Particularly, the CKD incidence rates were similar between the group with an eGFR of <60 mL/min and the group with diabetes and an eGFR of 60–89 mL/min without diabetes. The CKD incidence rate was higher in the group with diabetes with an eGFR of ≥90 mL/min than in the group with an eGFR of 60–89 mL/min without diabetes (Figure 2 and Figure 3).

**Figure 4:**
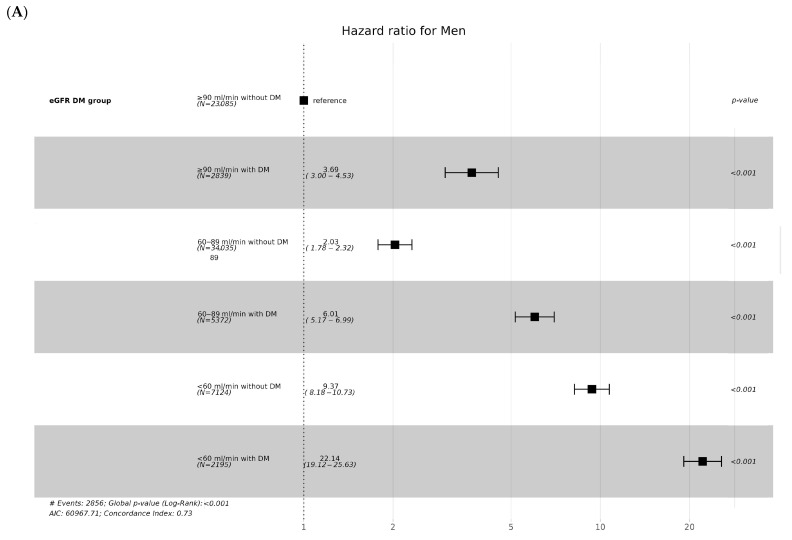

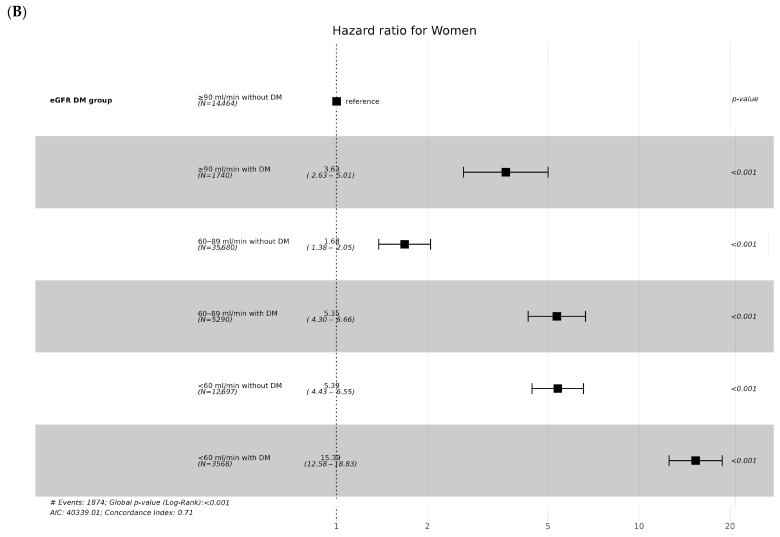
Hazard ratios (95% confidential interval) of incidence of CKD according to eGFR and Diabetes in men (**A**) and women (**B**).

The incidence of CKD was higher in men than in women in all groups classified based on eGFR and diabetes. Moreover, when both men and women had diabetes, the incidence and hazard ratio of CKD were higher compared to each eGFR group without diabetes (Table 2, Figure 4).

### 3.3. CKD Incidence according to Age and Sex

**Figure 5:**
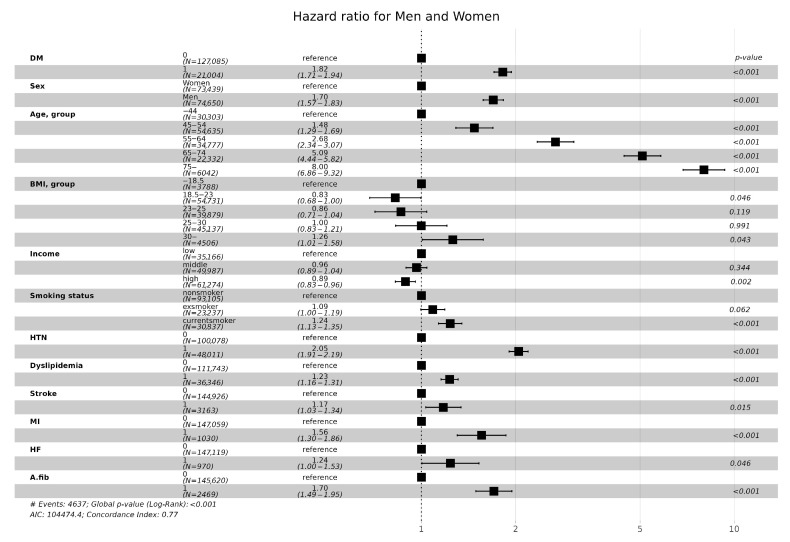
Hazard ratios (95% confidential interval) of incidence of CKD according to various clinical factors. Adjusted for sex and BMI, age, income, smoking, hypertension, dyslipidemia, ischemic stroke history, myocardial infarction history, heart failure, Atrial fibrillation, and flutter. Abbreviations: CKD, chronic kidney disease; BMI, body mass index; DM, diabetes mellitus; HTN, Hypertension; Stroke, ischemic stroke history; MI, myocardial infarction history; HF, heart failure; A.fib, Atrial fibrillation and flutter.

**Figure 6:**
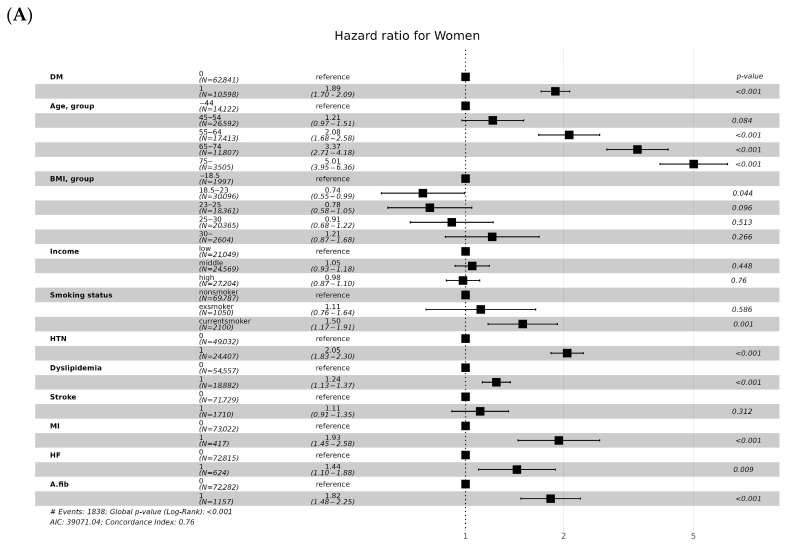

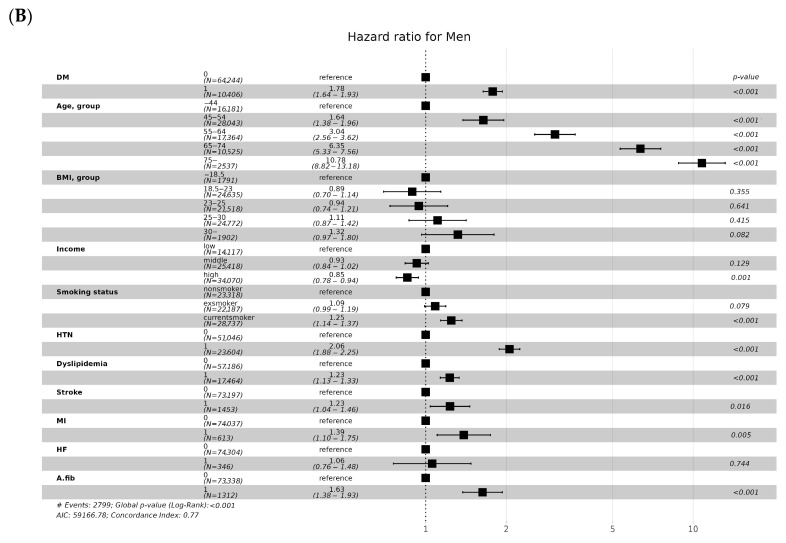
(**A**) Hazard ratios (95% confidential interval) of incidence of CKD according to various clinical factors in women. Adjusted for BMI, age, income, smoking, hypertension, dyslipidemia, ischemic stroke history, myocardial infarction history, heart failure, Atrial fibrillation, and flutter. (**B**) Hazard ratios (95% confidential interval) of incidence of CKD according to various clinical factors in men. Adjusted for BMI, age, income, smoking, hypertension, dyslipidemia, ischemic stroke history, myocardial infarction history, heart failure, Atrial fibrillation, and flutter. Abbreviations: CKD, chronic kidney disease; BMI, body mass index; DM, diabetes mellitus; HTN, Hypertension; Stroke, ischemic stroke history; MI, myocardial infarction history; HF, heart failure; A.fib, Atrial fibrillation and flutter.

Age was a strong factor in the incidence of CKD. the hazard ratio of CKD incidence was more than eight times higher in people over 75 years of age than in people under 44 years of age (Figure 5) In particular, the hazard ratio in CKD incidence for men under 44 years old and over 75 years old was 10 times the hazard ratio, but for women there was a difference of 5 times (Figure 6A,B). Men had a 1.7-fold higher risk of developing CKD than women (Figure 5). In men, ischemic stroke history affected CKD incidence (Figure 6A). In contrast, heart failure was associated with the risk of developing CKD in women (Figure 6B). In men, high income was associated with a lower risk of developing CKD compared to low income, but there was no difference in women (Figure 6A,B).

### 3.4. CKD Incidence according to Cardiovascular Risk 

Hypertension, dyslipidemia, stroke, myocardial infarction history, atrial fibrillation and flutter increased the risk of CKD incidence by 2.05, 1.23, 1.17, 1.56, 1.24, and 1.70 times, respectively (Figure 5).

## 4. Discussion

In this study, we confirmed the incidence of CKD according to eGFR among individuals who had undergone general national health checkups in South Korea. The main finding suggested that diabetes and age was highly associated with CKD incidence and the risk of development of CKD was higher in individuals with older age and lower eGFR value.

Due to the annual health checkup data, it is difficult to know whether the lowered eGFR may be temporarily decreased or due to CKD. eGFR can easily change due to dehydration, medication, etc. We could more accurately identify CKD by defining it as diagnosed by a clinician rather than simply defining it according to eGFR values. We included 25,584 (17.3%) participants with an eGFR <60 mL/min who were not diagnosed with CKD. During the follow up period, 2128 (8.3%) were diagnosed with CKD. This indicated that the eGFR value did not simply indicate CKD. Furthermore, in a study by Ryan et al., the clinical diagnosis rate of CKD was 26.5%, reporting that 74% of patients with CKD were undiagnosed [5]. From another perspective, it is highly likely that a significant number of CKD patients in Korea remain undiagnosed.

The effects of diabetes on renal function and the development and progression of CKD are well known. In diabetes, hyperfiltration injury, glycosylation end products, reactive oxygen specifications, various hormones, and cytokines cause diabetic nephropathy. In our study, the group with diabetes had a 1.82 times higher incidence of CKD than the group without diabetes. 

Older age was associated with a higher incidence of CKD. It has been well reported an increased CKD risk with age [6,7,8]. In addition, the prevalence of CKD in women is reported to be 1.76 times higher than that in men in Korea [9]. The absolute value of eGFR in women is lower than in men, while the rate of decline in eGFR is lower than in men [10]. In our study, compared to under 44 years of age, the risk of CKD in over 75 years of age was twice as high in men compared to women.

Our study showed a higher incidence of CKD in men than in women, even when other factors, including comorbidities, were corrected. However, in global (including Korea) studies on CKD prevalence, the prevalence of CKD is higher in women [11]. In a study on CKD prevalence in Korea involving 2356 individuals, the CKD prevalence between men and women was not significantly different (13.9% [women] vs. 13.5% [men]); however, the prevalence was higher in men aged <50 years, and the prevalence of CKD was higher in women over 50 years of age. They explaned the gender difference in CKD prevalence with age as follows; the increased prevalence of hypertension, diabetes, and BMI among men [3]. However, our study showed that women had a higher prevalence of diabetes (women vs. men, 14.4% vs. 13.9%, *p* = 0.006) and hypertension (women vs. men, 33.2% vs. 31.6%, *p* < 0.001, Supplementary Table S2). And there were more elderly patients among women. In addition, eGFR values were higher in men than in women. Based on these results, it could be expected that the incidence of CKD would be higher in women than in men, but our results showed a higher incidence in men. Although this is difficult to explain clearly, the rate of eGFR decline with age is greater in men than in women, so one would expect the incidence of CKD to be higher in men.

In baseline characteristics, the population of current smokers is lower in eGFR < 60 mL/min group. If our study population was divided by gender, 93.2% of the current smoker are men, but 6.8% of the current smoker among are women. In addition, the proportion of women in the eGFR < 60 mL/min group was 63.6%, which was higher than that of men. Among those in the <60 mL/min group, the number of women nonsmokers was high at 60.6% (Supplementary Table S3). Therefore, the proportion of current smokers with eGFR < 60 mL/min was low. Men and women demonstrated a high risk of developing CKD in current smokers (HR 1.24, men 1.25, women 1.50), which was consistent with the results of smoking as an independent risk factor for CKD incidence [12,13,14]. Smoking increases the risk of CKD through a proinflammatory state, oxidative stress, prothrombotic shift, endothelial dysfunction, glomerulosclerosis, and tubular atrophy. Our study showed a higher risk of developing CKD in women smokers, and similar results were found in a meta-analysis [13].

In our study, high-income men were associated with a lower risk of CKD. An imbalance in socioeconomic status has a negative effect on chronic diseases. This can be related to poor healthcare access in low-income individuals, poor lifestyle and nutrition, and jobs. In a study conducted in the United States, the higher the income, the lower the CKD prevalence [15]. In another study conducted in Korea, men and women in low-income groups were at high risk of developing CKD [16]. In the high income group, the risk of CKD tended to be low. Although this study was conducted by dividing the income level into ten quintiles, our study may be different because we divided the income into three quintiles. Furthermore, in our study, income was surveyed on a household basis. The income of a household is mainly composed of male income; therefore, women with low income are probably covered.

Hypertension is a well-known and strong risk factor for the development of CKD. Hypertension is transferred to intraglomerular capillary pressure, resulting in glomerular sclerosis and kidney injury. In our study, hypertension also increased the risk of developing CKD regardless of sex (HR 2.05, men 2.06, women 2.05). Dyslipidemia is also one of the predictors of progression for developing CKD [14,17]. In our study, Dyslipidemia had a similar risk of CKD incidence in men and women (HR 1.23, men 1.23, women 1.24).

CKD is one of the major complications following a myocardial infarction (MI) [14,18]. In our study, MI history in men and women increased the risk of development of CKD. CKD was more common in women with coronary artery disease [19]. Our study similarly showed a higher risk of developing CKD in women after MI (HR 1.56, men 1.39, women 1.93). Differences in the incidence of CKD in women and men with coronary artery disease have not been fully explained. Women with coronary artery disease are generally older and have more risk factors than men. Differences in treatment for women and men with MI may also cause CKD. Drugs at the same dose (fibrinolytic, contrast agent, ARB, etc.) may have a greater effect in women who usually have lower weight than in men. CKD and heart failure have a connection [20]. In more than 80,000 patients with heart failure, >63% had renal impairment [21]. Particularly, it acts as an independent predictor of rapid kidney function decline in the elderly aged >64 years [22]. The heart and kidneys play important roles in maintaining fluid homeostasis and normal blood pressure. Heart failure progresses and persists with CKD through reduced renal blood flow, renal hemodynamic impairment, and ischemic injury [23,24]. In our study, heart failure increased the risk of CKD only in women (HR 1.24, men 1.06 (no statistical significance), women 1.44). Direct studies on the difference in CKD incidence by sex in patients with heart failure are limited. McAlister et al. reported more women with CKD than men in patients with heart failure [20]. Women had a higher CKD incidence in a study comparing patients with left ventricular systolic dysfunction after acute MI [19]. Women with heart failure also have more common comorbidities than men [25]. MI can be explained similarly to why women have a high rate of CKD progression.

Ischemic stroke is a cardiovascular event, and stroke history is also a predictor of CKD progression [26,27,28]. After stroke, the neuroendocrine system, inflammatory and immune responses, etc., affect the brain–kidney interaction, which can lead to kidney dysfunction [29]. In our study, stroke was associated with a risk of CKD in men (HR 1.17, men 1.23, women 1.12 [no statistical significance]). However, in the study by Chwojnicki et al., CKD was higher in women than in men after an ischemic stroke [27]. A study comparing the incidence of end-stage renal disease (ESRD) after stroke also showed a high incidence of ESRD in both men and women [30]. Because studies on CKD incidence after stroke are limited, additional research on CKD incidence after stroke by sex is needed.

Atrial fibrillation and flutter can also increase the risk of developing CKD [31]. Activation of RASS acts as a major factor in the pathogenesis and progression of CKD and contributes to the development of atrial fibrillation and flutter. This association serves as an important link between atrial fibrillation, flutter, and CKD. Our study showed similar results to other studies (HR 1.70, men 1.63, women 1.82).

We could identify the limitations of this study as follows:(1) the diagnosis of CKD may have been overestimated in patients with comorbidities because they visit the hospital more, (2) because tests for albuminuria were not included in health checkups in Korea, CKD incidence according to albuminuria could not be analyzed, (3) because CKD incidence was identified through smoking habits and BMI during health checkups, changes in smoking habits and BMI after health checkups were not considered, (4) information on the duration of diabetes and the degree of control of diabetes was not obtained; therefore, their effect on CKD incidence was unknown, (5) comorbidities were being controlled to some extent, and information on the degree or sequelae was not obtained, (6) Peripheral vascular disease, a risk factor for CKD, was not included as a risk factor, (7) the eGFR value obtained from the health checkup may have had an error because it was a test result performed at multiple institutions, and (8) In accordance with Korea’s health examination regulations, eGFR is calculated using the MDRD formula during health examinations and data including eGFR values is provided. However, the MDRD formula requires further validation in Asian populations.

## 5. Conclusions

It was confirmed that old age and diabetes are important factors in the occurrence of CKD via the large-scale cohort data provided by the Korean NHIS. Additionally, it has been confirmed that as age increases, the risk of developing CKD in men increases compared to women. These results will be helpful in predicting risk groups for CKD and establishing strategies to lowering CKD incidence.

## Figures and Tables

**Table 1:** Participants Baseline characteristics by eGFR and Diabetes Mellitus.

Variables	eGFR and DM						
	≥90 mL/min/1.73 m^2^ w/o DM	≥90 mL/min/1.7 3 m^2^ with DM	60–89 mL/min/1.73 m^2^ w/o DM	60–89 mL/min/1.73 m^2^ with DM	<60 mL/min/1.73 m^2^ w/o DM	<60 mL/min/1.73 m^2^ with DM	*p*-Value
Total (n)	37,549	4579	69715	10,662	19,821	5763	
CKD (n)	419 (1.1%)	183 (4.0%)	1380 (2.0%)	620 (5.9%)	1268 (6.4%)	860 (14.9%)	<0.001
Sex							<0.001
Women	14,464 (38.5%)	1740 (38.0%)	35,680 (51.2%)	5290 (49.6%)	12,697 (64.1%)	3568 (61.9%)	
Men	23,085 (61.5%)	2839 (62.0%)	34,035 (48.8%)	5372 (50.4%)	7124 (35.9%)	2195 (38.1%)	
Age, group (years)							<0.001
40–44	14,580 (38.8%)	804 (17.6%)	13,617 (19.5%)	523 (4.91%)	740 (3.73%)	39 (0.68%)	
45–54	17,168 (45.7%)	2047 (44.7%)	29,263 (42.0%)	2749 (25.8%)	3134 (15.8%)	274 (4.75%)	
55–64	4807 (12.8%)	1295 (28.3%)	18,277 (26.2%)	4190 (39.3%)	5037 (25.4%)	1171 (20.3%)	
65–74	946 (2.52%)	408 (8.91%)	7812 (11.2%)	2883 (27.0%)	7461 (37.6%)	2822 (49.0%)	
≥75	48 (0.13%)	25 (0.55%)	746 (1.07%)	317 (2.97%)	3449 (17.4%)	1457 (25.3%)	
BMI, group (kg/m^2^)							<0.001
<18.5	163 (0.43%)	13 (0.28%)	1474 (2.12%)	98 (0.92%)	1741 (8.79%)	299 (5.19%)	
18.5–23	8010 (21.3%)	636 (13.9%)	29,647 (42.5%)	3152 (29.6%)	10,679 (53.9%)	2607 (45.3%)	
23–25	9982 (26.6%)	1043 (22.8%)	19,974 (28.7%)	3160 (29.6%)	4275 (21.6%)	1445 (25.1%)	
25–30	17,000 (45.3%)	2347 (51.3%)	17,610 (25.3%)	3869 (36.3%)	2983 (15.1%)	1328 (23.1%)	
≥30	2381 (6.34%)	539 (11.8%)	984 (1.41%)	382 (3.58%)	138 (0.70%)	82 (1.42%)	
Income, group							<0.001
Low	7923 (21.4%)	1038 (22.9%)	17,220 (25.0%)	2613 (24.8%)	5057 (25.8%)	1315 (23.0%)	
Middle	12,810 (34.6%)	1645 (36.3%)	23,728 (34.4%)	3647 (34.6%)	6418 (32.7%)	1739 (30.4%)	
High	16,338 (44.1%)	1848 (40.8%)	27,976 (40.6%)	4290 (40.7%)	8163 (41.6%)	2659 (46.5%)	
Smoking, group							<0.001
Non-smoker	19,777 (52.9%)	2490 (54.7%)	44,768 (64.6%)	6979 (65.9%)	14,808 (75.2%)	4283 (74.8%)	
Ex-smoker	7054 (18.9%)	900 (19.8%)	10,583 (15.3%)	1744 (16.5%)	2230 (11.3%)	726 (12.7%)	
Current-smoker	10,527 (28.2%)	1162 (25.5%)	13,897 (20.1%)	1870 (17.7%)	2662 (13.5%)	719 (12.6%)	
HTN	8064 (21.5%)	2429 (53.0%)	18,022 (25.9%)	6388 (59.9%)	8892 (44.9%)	4216 (73.2%)	<0.001
Dyslipidemia	6934 (18.5%)	2194 (47.9%)	13,841 (19.9%)	5199 (48.8%)	5301 (26.7%)	2877 (49.9%)	<0.001
Stroke	342 (0.91%)	130 (2.84%)	1013 (1.45%)	503 (4.72%)	708 (3.57%)	467 (8.10%)	<0.001
MI	125 (0.33%)	48 (1.05%)	300 (0.43%)	161 (1.51%)	228 (1.15%)	168 (2.92%)	<0.001
HF	87 (0.23%)	44 (0.96%)	265 (0.38%)	143 (1.34%)	246 (1.24%)	185 (3.21%)	<0.001
A. fib	387 (1.03%)	93 (2.03%)	828 (1.19%)	331 (3.10%)	565 (2.85%)	265 (4.60%)	<0.001
Death (n)	1195 (3.18%)	329 (7.18%)	3771 (5.41%)	1351 (12.7%)	4146 (20.9%)	1868 (32.4%)	<0.001
Creatinine (mg/dL)	1.0 ± 1.2	0.9 ± 1.0	1.1 ± 1.3	1.0 ± 1.1	1.2 ± 1.4	1.2 ± 1.2	<0.001
SBP (mmHg)	124.2 ± 14.7	126.9 ± 14.8	122.9 ± 15.3	127.0 ± 15.7	125.7 ± 16.7	129.3 ± 16.8	<0.001
DBP (mmHg)	78.0 ± 10.3	79.1 ± 9.9	76.6 ± 10.1	78.0 ± 10.0	76.9 ± 10.3	77.5 ± 10.3	<0.001

Data are expressed as mean ± standard deviation or number. Abbreviations: CKD, chronic kidney disease; eGFR, estimated glomerular filtration rate; BMI, body mass index; DM, diabetes mellitus; HTN, Hypertension; Stroke, ischemic stroke history; MI, myocardial infarction history; HF, heart failure; A.fib, Atrial fibrillation and flutter; SBP, systolic blood pressure; DBP, diastolic blood pressure.

**Table 2:** Comparison of CKD incidence in men and women according to eGFR.

Variables	eGFR and DM						
	≥90 mL/min/1.73 m^2^ w/o DM	≥90 mL/min/1.73 m^2^ with DM	60–89 mL/min/1.73 m^2^ w/o DM	60–89 mL/min/1.73 m^2^ with DM	<60 mL/min/1.73 m^2^ w/o DM	<60 mL/min/1.73 m^2^ with DM	*p*-Value
Total (n)	37,549	4579	69,715	10,662	19,821	5763	
CKD	419 (1.1%)	183 (4.0%)	1380 (2.0%)	620 (5.9%)	1268 (6.4%)	860 (14.9%)	<0.001
Sex							
Women (n)	14,464	1740	35,680	5290	12,697	3568	
CKD	123 (0.9%)	53 (3.0%)	509 (1.4%)	235 (4.4%)	548 (4.3%)	406 (11.4%)	<0.001
Men (n)	23,085	2839	34,035	5372	7124	2195	
CKD	296 (1.3%)	130 (4.6%)	871 (2.6%)	385 (7.2%)	720 (10.1%)	454 (20.7%)	<0.001

Abbreviations: CKD, chronic kidney disease; eGFR, estimated glomerular filtration rate; DM, diabetes mellitus.

## Data Availability

The original contributions presented in the study are included in the article/Supplementary Materials, further inquiries can be directed to the corresponding authors.

## Supplementary Materials

The following supporting information can be downloaded at: https://www.mdpi.com/article/10.3390/jcm13082164/s1, Table S1: Participants baseline characteristics by estimated glomerular filtration rate group; Table S2. Participants baseline characteristics by Sex; Table S3. Smoking status according to Sex and eGFR.
