# Supplementary material for: The Factors Influencing Chronic Kidney Disease Incidence: Database from the Korean National Health Insurance Sharing Service (NHISS)
Source: J Clin Med. 2024 Apr 9;13(8):2164. doi: 10.3390/jcm13082164 (PMC11050717; doi:10.3390/jcm13082164)
Supplement: Supplementary file 1 [file jcm-13-02164-s001.zip › jcm-2907886-supplementary.pdf]

Journal of Clinical Medicine

MDPI

Supplementary Materials

Table S1. Participants baseline characteristics by estimated glomerular filtration rate group.

Variables	eGFR <60 ml/min/1.73m²	eGFR 60–89 ml/min/1.73m2	eGFR ≥90 ml/min/1.73m²	p-value
Total (n)	25,584	80,377	42,128	
CKD (n)	2128 (8.3%)	2000 (2.5%)	602 (1.4%)	<0.001
Sex				<0.001
Women	16,265 (63.6%)	40,970 (51.0%)	16,204 (38.4%)	
Men	9,319 (36.4%)	39,407 (49.0%)	25,924 (61.5%)	
Age, group (years)				<0.001
40–44	779 (3.0%)	14,140 (17.6%)	15,384 (36.5%)	
45–54	3408 (13.3%)	32,012 (39.8%)	19215 (45.6%)	
55–64	6,208 (24.3%)	22,467 (28.0%)	6,102 (14.5%)	
65–74	10,283 (40.2%)	10,695 (13.3%)	1354 (3.2%)	
≥75	4906 (19.2%)	1,063 (1.3%)	73 (0.2%)	
BMI, group (kg/m²)				<0.001
<18.5	2040 (8.0%)	1572 (2.0%)	176 (0.4%)	
18.5–23	13,286 (51.9%)	32,799 (40.8%)	8646 (20.5%)	
23–25	5720 (22.4%)	23,134 (28.8%)	11,025 (26.2%)	
25–30	4311 (16.9%)	21,479 (26.7%)	19,347 (45.9%)	
≥30	220 (0.9%)	1366 (1.7%)	2920 (6.9%)	
Income, group				<0.001
Low	6372 (25.1%)	19,833 (25.0%)	8961 (21.5%)	
Middle	8157 (32.2%)	27,375 (34.4%)	14,455 (34.7%)	
High	10,822 (42.7%)	32,266 (40.6%)	18,186 (43.7%)	
Smoking, group				<0.001
Nonsmoker	19,091 (75.1%)	51,747 (64.8%)	22,267 (53.1%)	
Ex-smoker	,956 (11.6%)	12,327 (15.4%)	7954 (19.0%)	
Current smoker	3381 (13.3%)	15,767 (19.7%)	11,689 (27.9%)	
DM	5763 (22.5%)	10,662 (13.3%)	4579 (10.9%)	<0.001
HTN	13,108 (51.2%)	24,410 (30.4%)	10,493 (24.9%)	<0.001
Dyslipidemia	8178 (32.0%)	19,040 (23.7%)	9128 (21.7%)	<0.001
Stroke	1175 (4.6%)	1516 (1.9%)	472 (1.1%)	<0.001
MI	396 (1.6%)	461 (0.6%)	173 (0.4%)	<0.001
HF	431 (1.7%)	408 (0.5%)	131 (0.3%)	<0.001
A. fib	830 (3.2%)	1159 (1.4%)	480 (1.1%)	<0.001
Death (n)	6014 (23.5%)	5122 (6.4%)	1524 (3.6%)	<0.001
Creatinine (mg/dL)	1.2 ± 1.3	1.1 ± 1.3	1.0 ± 1.2	<0.001
SBP (mmHg)	126.5 ± 16.8	123.4 ± 15.4	124.5 ± 14.7	<0.001
DBP (mmHg)	77.0 ± 10.3	76.8 ± 10.1	78.1 ± 10.3	<0.001

Data have been expressed as mean ± standard deviation or number. Abbreviations: CKD, chronic kidney disease; eGFR, estimated glomerular filtration rate; BMI, body mass index; DM, diabetes mellitus; HTN, Hypertension; Stroke, ischemic stroke history; MI, myocardial infarction history; HF, heart failure; A.fib, atrial fibrillation and flutter; SBP, systolic blood pressure; DBP, diastolic blood pressure.

Table S2. Participants baseline characteristics by Sex.

Variables	Men (%)	Women (%)	p-value
Total (n)	74,650	73,439	

CKD (n)	2856 (3.8%)	1874 (2.6%)	<0.001
Age, group (years)			<0.001
40-44	16,181 (21.7%)	14,122 (19.2%)	
45-54	28,043 (37.6%)	26,592 (36.2%)	
55-64	17,364 (23.3%)	17,413 (23.7%)	
65-74	10,525 (14.1%)	11,807 (16.1%)	
≥75	2537 (3.4%)	3505 (4.8%)	
BMI, group (kg/m^2)			<0.001
<18.5	1791 (2.4%)	1997 (2.7%)	
18.5-23	24,635 (33.0%)	30,096 (41.0%)	
23-25	21,518 (28.8%)	18,361 (25.0%)	
25-30	24,772 (33.2%)	20,365 (27.7%)	
≥30	1902 (2.5%)	2604 (3.5%)	
Income, group			<0.001
Low	14,117 (18.9%)	21,049 (28.7%)	
Middle	25,418 (34.0%)	24,569 (33.5%)	
High	34,070 (45.6%)	27,204 (37.0%)	
Smoking, group			<0.001
Non-smoker	23318	69,787	
Ex-smoker	22187	1050	
Current-smoker	28737	2100	
DM	10,406 (13.9%)	10,598 (14.4%)	0.006
HTN	23,604 (31.6%)	24,407 (33.2%)	<0.001
Dyslipidemia	17,464 (23.4%)	18,882 (25.7%)	<0.001
Stroke	1453 (1.9%)	1710 (2.3%)	<0.001
MI	613 (0.8%)	417 (0.6%)	<0.001
HF	346 (0.5%)	624 (0.8%)	<0.001
A. fib	1312 (1.8%)	1157 (1.6%)	<0.001

Abbreviations: CKD, chronic kidney disease; BMI, body mass index; DM, diabetes mellitus; HTN, Hypertension; Stroke, ischemic stroke history; MI, myocardial infarction history; HF, heart failure; A.fib, atrial fibrillation and flutter.

Table S3. Smoking status according to Sex and eGFR.

Variables		eGFR			p-value
	**Total**	**<60 ml/min/1.73m^2**	**60–89 ml/min/1.73m2**	**≥90 ml/min/1.73m^2**	
Nonsmoker	93,105				<0.001
Women	69,787 (74.6%)	15,411 (16.6%)	39,043 (41.9%)	15,333 (16.5%)	
Men	23,318 (33.4%)	3680 (4%)	12,704 (13.6%)	6934 (7.4%)	
Exsmoker	23,237				<0.001
Women	1050 (4.5%)	225 (1%)	561 (2.4%)	264 (1.1%)	
Men	22,187 (95.5%)	2731 (11.8%)	11,766 (50.6%)	7690 (33.1%)	
Current smoker	30,837				<0.001
Women	2100 (6.8%)	521 (1.7%)	1066 (3.5%)	513 (1.7%)	
Men	28,737 (93.2%)	2860 (9.3%)	14,701 (47.7%)	11,176 (36.2%)	

Abbreviations: eGFR, estimated glomerular filtration rate.
